# 80276 Use of Live Community Events on Facebook to Share Health and Clinical Research Information with the Community: An Exploratory Study

**DOI:** 10.1017/cts.2021.538

**Published:** 2021-03-30

**Authors:** Jinhee Cha, Ian West, Tabetha A. Brockman, Miguel Valdez Soto, Elisia L. Cohen, Joyce (Joy) E. Balls-Berry, Milton (Mickey) Eder

**Affiliations:** 1 University of Minnesota, Center and Translational Science Institute; 2 Mayo Clinic, Center for Clinical and Translational Sciences; 3 Washington University Saint Louis, Knight ADRC

## Abstract

ABSTRACT IMPACT: We review our strategy to use live community events on Facebook to share health and clinical research information and share further steps to increase engagement. OBJECTIVES/GOALS: To describe the use of live community events to enhance communication about clinical and health research through a Facebook platform (MN Research Link) with diverse social media users. The project identified variables associated with video engagement and strategic implications. METHODS/STUDY POPULATION: From June 2019 to November 2020 we streamed 31 events on the MN Research Link Facebook public page. Events highlighted different investigators’ clinical and health research in the areas of mental health, health and wellness, chronic diseases and immunology/infectious diseases. Facebook analytics were used to determine the number of views, total minutes viewed, average video watch time, and audience retention. Engagement score was calculated as the total number of interactions (likes, shares, and comments) divided by total number of followers (N=1437), expressed as a percentage RESULTS/ANTICIPATED RESULTS: Events averaged 24secs/16 min (SD=0.4). A mean of 1.61 (SD=1.28) followers viewed the events live but an average of 417.52 (SD=793.50) followers viewed after the event posted. The average engagement score was 1.1%. Mean total minutes viewed for all 31 videos was 253.5 (SD= 437.6). Viewers spent an average of 17 seconds (SD=0.01) watching each piece of video content. On average 28 followers viewed the events for at least 1-minute event (SD= 48.7). Audience retention at the halfway point for each video was 15.74% (SD=0.19). DISCUSSION/SIGNIFICANCE OF FINDINGS: Results suggest that novel approaches are necessary for active engagement. Promotion of live events is recommended to increase participation and length of engagement. Prior length of engagement (average 17 seconds), suggests refining video introduction will increase engagement.

